# Associations of parenting stress with coping strategies, dental fear, and behaviour during dental treatment in school-aged children: a cross-sectional study

**DOI:** 10.1186/s12903-025-07279-2

**Published:** 2025-12-20

**Authors:** Baranya Shrikrishna Suprabha, Harshitha Sharma, Ramya Shenoy, Arathi Rao, Kunnumal Thekkeveetil Shwetha, Raghbir Kaur

**Affiliations:** 1 Department of Pediatric and Preventive Dentistry, Manipal College of Dental Sciences Mangalore, Manipal Academy of Higher Education, Manipal, India; 2 Department of Public Health Dentistry, Manipal College of Dental Sciences Mangalore, Manipal Academy of Higher Education, Manipal, India; 3 Anirvedha Resource Centre for Psychological Well-Being, Mangalore, India; 4 Attending Faculty, Yale New Haven Hospital, New Haven, CT USA

**Keywords:** Parenting stress, Child, Coping strategies, Dental fear, Behaviour, Dentistry

## Abstract

**Background:**

Parenting stress can affect coping strategies and behaviour problems among children in nondental settings.

**Objective:**

To evaluate the associations of parenting stress with children’s coping strategies, dental fear, and behaviour during dental treatment in school-aged children.

**Methods:**

This cross-sectional study included *n* = 168 children aged 8–12 years who required dental treatment under local anaesthesia. The accompanying parents completed the Parental Stress Scale (PSS), and the children completed the Dental Coping Questionnaire (DCQ) and the Children’s Fear Survey Schedule-Dental Subscale (CFSS-DS) prior to the start of the treatment procedure. The behaviour of the children was evaluated during their treatment period using Frankl’s behaviour rating scale. The associations of parenting stress with the measured parameters were analysed via chi-square test, Spearman’s correlation, Kruskal‒Wallis test, Mann‒Whitney U test, and generalised linear model, with statistical significance set at *p* ≤ 0.05.

**Results:**

No significant correlation between the PSS score and the percentage frequency of use of destructive, external, and internal coping strategies was detected. There was no significant difference in the PSS scores of parents between children who had no clinical dental fear (CFDSS score < 32), borderline dental fear (CFSS-DS score 32–38) and clinical dental fear (CFSS-DS score ≥ 39) or between children with uncooperative (Frankl scores 1 and 2) and cooperative behaviour (Frankl scores 3 and 4). No significant associations between PSS scores and the study variables were detected via the generalised linear model.

**Conclusion:**

Parenting stress does not significantly influence children’s utilisation of coping strategies, dental fear or behavioural responses during dental procedures among children aged 8–12 years.

## Introduction

Disruptive and uncooperative behaviour by children during dental treatment can delay the treatment procedure or render treatment delivery impossible [1, 2]. While dental fear is a key contributor to uncooperative behaviour in children, psychosocial and environmental factors also play important roles [1, 3]. Dental fear refers to unpleasant emotions connected to dental treatment. It is a response driven by emotions to specific threatening factors in the dental treatment setting [1]. A review of studies in 12 different populations revealed a prevalence of dental fear between 5.7% and 19.5% in the 3–18-year age group, whereas a recent meta-analysis revealed pooled prevalences of 36.5%, 25.8% and 13.3% for preschoolers, school children and adolescents, respectively [1, 4]. Painful and unpleasant stimuli during past dental and medical experiences, parental anxiety, parenting style, and a child’s own personality traits can influence the dental fear of the child [1, 3, 5, 6].

Coping is “the cognitive and behavioural efforts used to manage external and/or internal stressful demands that are appraised to exceed the resources of the person“ [7]. This refers to strategies to manage challenging situations and handle their negative outcomes [8] The selection of coping strategies during dental treatment is shaped by the level of dental anxiety and prior experience with pain [9]. Gaining insight into the coping mechanisms that children use and how helpful they perceive them to be can assist dentists in encouraging the effective application of these strategies, thereby fostering a more positive treatment experience [10].

Parental interactions with the child and presence during treatment significantly influence the child’s dental fear and behaviour in the clinical setting [11]. Parental anxiety, particularly maternal anxiety, can subtly influence a child’s dental behaviour, with highly anxious mothers often exerting a negative impact [12]. Parenting styles such as authoritative (balanced warmth and control), authoritarian (high control and low responsiveness) or permissive (high responsiveness and low control) can affect social skills, coping ability, and the ability to deal with stressful situations such as dental treatment [5, 13].

Stress is “a particular relationship between an individual and the environment that is appraised by the individual as exceeding personal resources and endangering well-being” [7]. Parenting stress refers to the feelings of distress or discomfort that arise from the challenges and demands involved in fulfilling the role of a parent [14]. Parenting involves a variety of stresses in child rearing, such as managing a child’s behaviour, financial issues, health concerns, and academic pursuits [15]. Parents’ responses to their children’s negative emotions may differ; they can be either supportive or non-supportive. Parents’ supportive responses help their children comprehend and cope with emotional situations. Non-supportive responses, such as dismissing the child’s emotional experience, disciplining the child, or being alarmed by the child’s conduct, may be ineffective in helping the child cope [16]. Greater parenting stress is associated with authoritarian parenting behaviour, characterised by negative interactions and less involvement with the child over time [17, 18]. As a result, the child’s capacity to cope with new and stressful situations is impaired [17]. Previous studies conducted in nondental settings have shown the influence of parenting stress on the coping strategies and behaviour problems of children [17, 19].

Considering the available evidence related to the role of parents, particularly parenting stress in the use of coping strategies and the behaviour of children in everyday situations, we considered it necessary to explore the influence of parenting stress on the coping strategies, fear and behaviour of children in dental situations. An understanding of such an association in the dental context would facilitate the effective implementation of behaviour guidance strategies for children during dental treatment, involving parents. Given the limited literature related to the role of parenting stress on the child’s coping strategies, fear and behaviour in a dental situations, the objective of our study was to assess the association of parenting stress with children’s dental coping strategies, along with the secondary objectives of assessing the associations of parenting stress with dental fear and behaviour during the dental treatment of school-aged children. The null hypothesis was that there is no association of parenting stress with the use of dental coping strategies, dental fear or behaviour of children during dental treatment.

## Methods

This cross-sectional study was conducted at the Paediatric and Preventive Dentistry Department of a teaching dental institution in Mangalore, a southern Indian city, from June 2021 to May 2022.

### Sampling and sample size

A convenience sample of all eligible children who visited the department clinic for dental treatment over a 12-month period was included after screening for inclusion and exclusion criteria.

Children aged 8–12 years with previous experience with dental treatment under local anaesthesia were included. In addition, children were required to have a treatment indication under local anaesthesia, such as restoration or pulp therapy or extraction. Children with developmental delay and medically compromised children were excluded.

The sample size was calculated as 84, assuming no correlation, R_0_ = 0.0 and R_1_ = 0.3 at a 95% confidence level and 80% power. To control for design effects due to variability in the data caused by confounding variables, the sample size was doubled to 168 [20].

### Ethical considerations

The study procedure was explained to the parents via a parent information sheet, and a verbal and written informed consent was obtained. The child’s written assent was obtained after the study procedure was explained via a participant information sheet. The participating children and parents were informed that the treatment procedure would be video recorded to help the investigators analyse behaviour during dental treatment and were assured that the confidentiality of all records related to the study would be maintained, via the parent and child participant information sheet. They were also assured that there was no pressure on them to answer the questionnaire and that the child’s treatment did not depend on their questionnaire responses. The study was conducted with the approval of the institutional ethics committee (Protocol reference no. 20019).

### Data collection

The parents of the participating children were administered a questionnaire on demographic details such as the age and sex of the parent‒child dyad and parenting stress using Parenting Stress Scale (PSS), while the children completed a questionnaire that consisted of the Dental Cope Questionnaire (DCQ) for dental coping and the Children’s Fear Survey Schedule-Dental Subscale (CFSS-DS) for dental fear before the commencement of the dental treatment procedure. The parents and children were introduced briefly to the questionnaire and shown how to respond, with an example mock-up question. Incompletely completed questionnaires were returned to the participants with a request to complete them. To minimise social desirability bias, participant anonymity and confidentiality were ensured. The study participants were blinded to the study hypothesis. A rapport was built by the investigator who gave out the questionnaire with the parents and their children, who ensured that there was no pressure on them to answer the questionnaire and that the child’s treatment did not depend on their questionnaire responses. Parents and children answered the questionnaire in a relaxed atmosphere and participants were encouraged to provide honest answers. Five subject experts confirmed the content validity related to clarity, comprehensiveness and relevance, and five parents who were not part of the study, before the questionnaire was administered to the study participants.

#### Assessment of parenting stress

The PSS is a self‑reported scale comprising 18 questions corresponding to blissful, or optimistic, and pessimistic aspects of parenthood [21]. The scale consists of two subconstructs: the parenting stress subscale (PS), which captures dimensions related to parenting stressors and perceived lack of control, and the lack of parental satisfaction subscale (LPS), which reflects dissatisfaction with the parenting role and a diminished sense of parental rewards. The reliability and validity of the PSS has been tested in Indian populations, showing high internal consistency (Cronbach’s α = 0.915–0.923) and test‒retest reliability [intraclass correlation coefficient (ICC) = 0.820–0.987] [22, 23].The parent was required to answer each question on a five‑point Likert scale: strongly disagree (1), disagree (2), undecided (3), agree (4), and strongly agree (5). LPS items were reverse scored as follows: (1 = 5) (2 = 4) (3 = 3) (4 = 2) (5 = 1). All the item scores were then summed to obtain the parenting stress score to obtain scores ranging from 18-90. Higher scores correspond to higher levels of parenting stress [24].

#### Assessment of dental coping in children

The DCQ, a 15-item modified version of the Kidcope questionnaire, was administered [25]. The coping strategies that the child had adapted when the child perceived pain or discomfort during earlier dental treatment procedures were revealed with a “yes” or “no” response (Scale A). The children were also asked about the effectiveness of the adapted coping strategies with the following question, “When yes, does it work? “, and the responses were given on a scale of “not at all”, “a little”, or “a lot” (Scale B). The coping strategies are divided into three categories: destructive, external and internal. Destructive coping strategies are maladaptive responses leading to negative dental experiences or avoidance of dental treatment [26]. External coping strategies are behavioural in nature and involve the use of external help from a person or a tool to positively cope with dental treatment. Internal coping strategies are thought-based and alleviate unpleasant emotions related to the dental treatment [9]. The percentage frequency of the use of the coping strategies and their helpfulness was obtained. According to an earlier study, the scale has moderate internal consistency (Cronbach’s α = 0.61) [9].

#### Assessment of dental fear in children

The CFSS-DS, which includes items specific to dental fear, was used [27]. The scale consists of 15 items related to various aspects of dental treatment. Each item was scored on a 5-point Likert scale ranging from 1 (not afraid at all) to 5 (very afraid). The total scores thus ranged from 15 to 75. The scores were categorised as no clinical dental fear (nonclinical range) (CFSS-DS score < 32), borderline dental fear (borderline range) (CFSS-DS score 32–38) and clinical dental fear (clinical range) (CFSS-DS score ≥ 39) [28, 29]. The reliability of this scale has been tested in the Indian population and has been shown to be very good (Cronbach’s α = 0.92) [30].

#### Assessment of the children’s behaviour

The indicated dental treatment procedure was performed on the children under local anaesthesia. During treatment, typical behaviour guidance techniques such as empathic personalised communication, tell-show-do, and positive reinforcements were used [31].

The dental behaviour was rated using Frankl’s behaviour rating scale, which categorises the child’s behaviour during dental treatment in the dental clinic into four categories: rating 1-definitely negative, rating 2-negative, rating 3-positive and rating 4-definitely positive [32]. Two investigators independently scored the behaviour by reviewing video recordings of the dental treatment procedure. The child’s behaviour was observed throughout the treatment to assess the behaviour. Behaviour rating was performed by observing the child’s behaviour between the time the child was seated in the dental chair until the dental treatment procedure was completed. The score representing the most negative behaviour of the child (peak behaviour score) during the observation period was considered [33]. The examiners were calibrated by independently rating the behaviour of 20 participants not part of the study to obtain more than 90% concurrence. During the study, the inter-examiner and intra-examiner reliability (rated twice, at an interval of two weeks) of both examiners were determined by rating the behaviour of 20 randomly selected participants.

Before the study, the test-retest reliability of the CFSS-DS, DCQ, and PSS scale was evaluated by having 20 children and their parents (not included in the final sample) complete the questionnaire twice, with a two-week gap between their responses. ICC and Cohen’s kappa values were calculated to determine the consistency of the responses. A two-way random effects model with absolute agreement was chosen for estimating the ICC. This reliability check aimed to confirm that parents and children comprehended the questionnaire and responded to it consistently.

### Statistical analysis

IBM SPSS software for Windows, version 29 (IBM Corp., Armonk, NY, USA), was used for data analysis. Descriptive statistics were obtained. Nonparametric tests were applied to determine the relationships between parenting stress and the study variables. To determine the relationships among the factors, generalised linear model regression analysis was performed, with demographic and study variables as dependent factors and parenting stress as an independent factor. The statistical significance was at *p* ≤ 0.05.

## Results

**Fig. 1:**
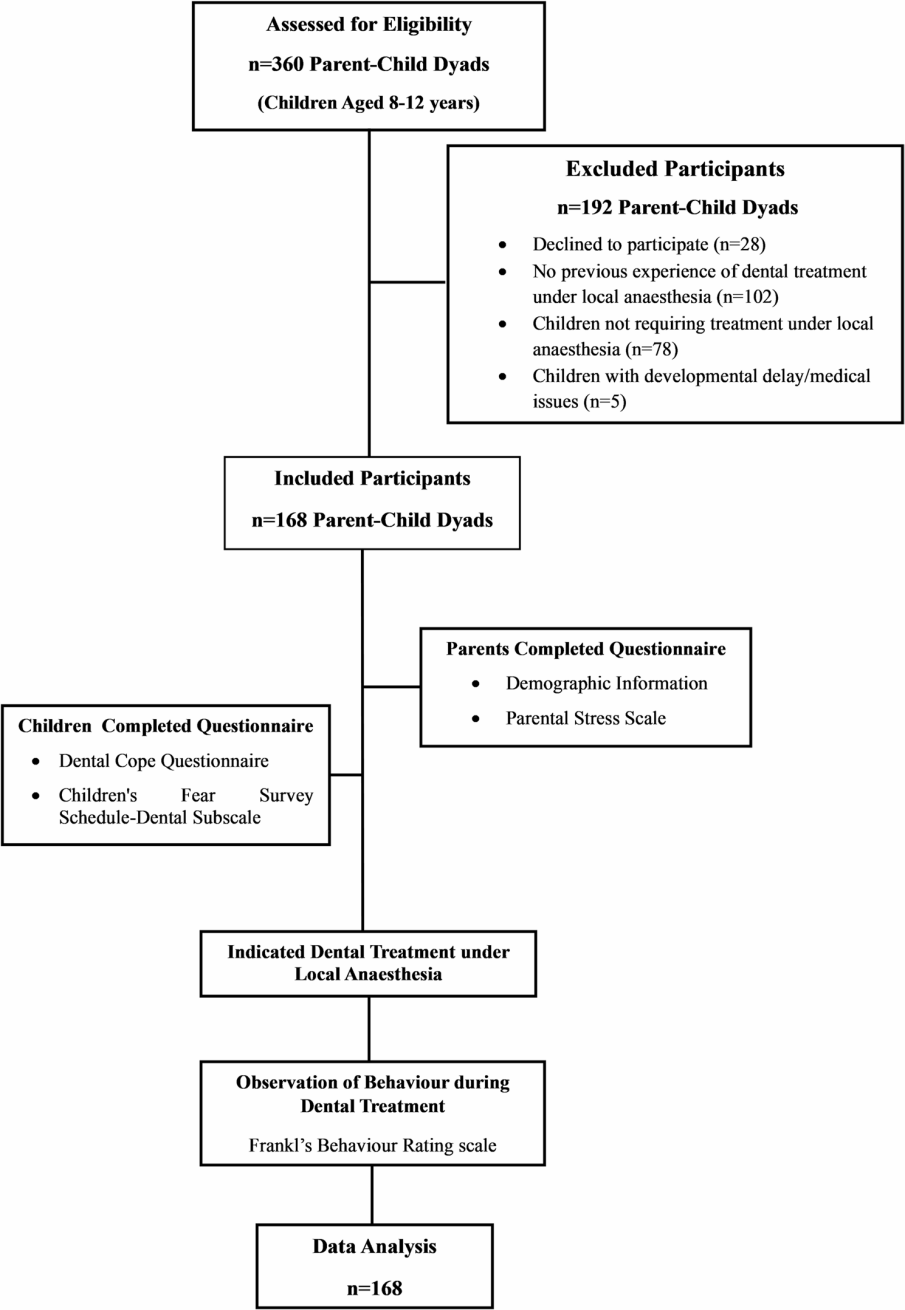
Flow Diagram showing the pathway for participant involvement in the study

All 168 parent‒child dyads returned completed questionnaires (Fig. 1). The mean age of the parents was 34.9 ± 4.08 years (range 20–45 years), whereas the mean age of the children was 9.07 ± 1.68 years (range 8–12 years). Among the parents who participated in the study, 62 (36.9%) were fathers, and 106 were mothers (63.1%). Among the child participants, 100 (59.5%) were boys, and 68 (40.5%) were girls. For dental treatment under local anaesthesia, 126 (85%) children received infiltration, and the remaining 42 (25%) received inferior alveolar nerve block. The treatments provided included 37 restorations (22%), 60 pulp therapies (35.8%) and 71 tooth extractions (42.2%).

The ICC was used to determine the test-retest reliability of the PSS scale and was rated as excellent (ICC = 0.92). The average PSS score of the sample was 35.48 ± 3.72. The two subscales of the PSS had mean values of 27.19 ± 3.47 (PS) and 8.29 ± 0.84 (LPS). Table 1 shows the frequency distributions of the PSS scores given by the parent participants. Most of the parents had high satisfaction scores on the LPS subscale. Most of the parents strongly agreed that “Caring for my child sometimes takes more time and energy than I have to give” and “I sometimes worry whether I am doing enough for my child”, while the response was “strongly disagree” by most parents for items such as “The major source of stress in my life is my child”; “Having a child leaves little time and flexibility in my life”; and “Having a child has been a financial burden”.

**Table 1:** Frequency distribution of parental stress scale (PSS) scores

Item Number	ITEM	Response				
		1	2	3	4	5
		N (%)	N (%)	N (%)	N (%)	N (%)
Parental Stress subscale (PS)						
3	Caring for my child sometimes takes more time and energy than I must give	1 (0.6)	1 (0.6)	-	39 (23.2)	127 (75.6)
4	I sometimes worry whether I am doing enough for my child	1 (0.6)	2 (1.2)	-	60 (35.7)	105 (62.5)
9	The major source of stress in my life is my child	84 (50.0)	72 (42.9)	9 (5.4)	2 (1.2)	1 (0.6)
10	Having a child leaves little time and flexibility in my life	98 (58.3)	54 (32.1)	12 (7.1)	2 (1.2)	2 (1.2)
11	Having a child has been a financial burden	107 (63.7)	56 (33.3)	1 (0.6)	2 (1.2)	2 (1.2)
12	It is difficult to balance different responsibilities because of my child	3 (1.8)	7 (4.2)	62 (36.9)	84 (50.0)	12 (71.)
13	The behaviour of my child is often embarrassing or stressful to me	60 (35.7)	73 (43.5)	16 (9.5)	18 (10.7)	1 (0.6)
14	If I had it to do over again, I might decide not to have child	19 (11.3)	61 (36.3)	57 (33.9)	23 (13.7)	8 (4.8)
15	I feel overwhelmed by the responsibility of being a parent	73 (43.5)	69 (41.1)	20 (11.9)	1 (0.6)	5 (3.0)
16	Having a child has meant having too few choices and too little control over my life	6 (3.6)	19 (11.3)	80 (47.6)	37 (22.0)	26 (15.5)
Lack of Parental Satisfaction subscale (LPS)-reversely scored items						
1	I am happy in my role as a parent	-	-	-	- -	168 (100.0)
2	There is little or nothing I wouldn’t do for my child if it was necessary	-	1 (0.6)	-	9 (5.4)	158 (94.0)
5	I feel close to my child	-	-	-	9 (5.4)	159(94.6)
6	I enjoy spending time with my child	2 (1.2)	-	-	-	166 (98.8)
7	My child is an important source of affection for me.	-	-	-	-	168 (100.0)
8	Having children gives me a more certain and optimistic view of the future.	-	-	-	1 (0.6)	167 (99.4)
17	I am satisfied as a parent	-	-	-	-	168 (100.0)
18	I find my child enjoyable		-	-	-	168 (100.0)

1=Strongly disagree; 2=Disagree; 3=Undecided; 4=Agree; 5= Strongly Agree

The test-retest reliability of the DCQ indicated substantial to almost perfect agreement (κ values for the items ranged from 0.64 to 1). Table 2 shows the frequency distribution of the coping strategies by children according to the DCQ. Among the destructive strategies, the most used strategy was “I close my mouth” (38.1%). Asking the dentist what he is doing (70.2%) and telling the dentist (77.4%) were the most used external strategies. Among the internal strategies, the most used was “think it is good for my teeth” (83.9%). Each of the 168 participants used at least one internal coping strategy, only 90 participants used destructive coping strategies, and 158 participants used at least one external coping strategy. Overall, the mean percentage of participants who used coping strategies was 48.17 ± 11.34%.

**Table 2:** Frequency distribution of strategies used by the children for coping and their helpfulness according to the dental Cope questionnaire (DCQ)

Scale A		Scale B: When yes, does it help?	
When I am in pain at the dentist,	Yes	Helpful	Not Helpful
	N (%)	N (%)	N (%)
I. Destructive Strategies			
I get angry at mum and dad	2(0.2)	0 (0)	2 (100)
I think of a reason to sneak out	37 (22.0)	5 (13.5)	32 (86.5)
I close my mouth	64 (38.1)	40 (62.5)	24 (37.5)
I get angry at the dentist	36 (21.4)	8 (22.2)	28 (77.8)
II. External			
I ask the dentist what he is doing	118 (70.2)	63 (53.4)	55 (46.6)
I look at the mirror	63 (37.5)	50 (79.4)	13 (20.4)
I tell the dentist	130 (77.4)	78 (60.0)	52 (40.0)
I like it to have friends with me	-	0 (0)	168 (100.0)
I like it when the nurse holds my hand	33 (19.6)	11 (33.3)	22 (66.7)
III. Internal Strategies			
I think of other things	88 (52.4)	77 (87.5)	11 (12.5)
I tell myself it will be over soon	69 (41.1)	57 (82.6)	12 (17.4)
I do what dentist tells me to do	138 (82.1)	18 (13.1)	120 (86.9)
I think it is good for my teeth	156 (92.9)	13 (8.3)	143 (91.7)
I think it is my own fault I have cavities	75 (44.6)	68 (90.7)	7 (9.3)
I think it is part of dentistry	141 (83.9)	18 (12.8)	123 (87.2)

Table 3 shows the descriptive statistics for the coping strategies and their perceived helpfulness. Overall, internal strategies were the most used and were perceived as “helpful” by the smallest number of participants. However, three internal coping strategies, “I tell myself it will be over soon” (82.6%), “I think of other things” (87.5%), and “I think it is my own fault I have cavities” (90.7%), were perceived as more helpful strategies (Table 2). On the other hand, although less frequently used, destructive strategies were found to be helpful by more participants.

**Table 3:** Spearman’s correlations (r_s_) of PSS scores with percentage frequency of coping strategy use and their perceived helpfulness

Coping Strategy		Destructive	External	Internal	Total
Percentage frequency of use	**Mean ± SD**	20.68 ± 24.67	40.95 ± 18.02	62.16 ± 15.88	48.17 ± 11.34
	**Median (IQR)**	25.00 (25.00)	40.00 (20.00)	57.14 (14.31)	46.17 (13.33)
	**r**_**s**_	0.01	−0.03	0.03	−0.02
	***p*** **value**	0.902	0.731	0.748	0.789
	**N**	168	168	168	168
Percentage frequency of helpfulness	**Mean ± ** **SD**	51.67 ± 40.47	58.38 ± 39.90	28.39 ± 25.30	43.28 ± 23.60
	**Median (IQR)**	50.00 (100)	50 (69.75)	25.00 (40.00)	42.86 (29.15)
	**r**_**s**_	−0.13	0.04	0.08	−0.01
	***p*** **value**	0.215	0.646	0.314	0.913
	**N**	90	168	158	168

*SD* Standard deviation, *IQR* Interquartile range, *N* Total, *p* > 0.05 = not significant, indicating no significance for all correlations

The Shapiro‒Wilk test results for the normality of the data were significant; hence, nonparametric tests were used to analyse the associations between the study variables. Spearman’s correlation coefficient test revealed no significant correlation between the percentage frequency of use or the helpfulness of dental coping strategies and PSS scores (Table 3). A significant but negative correlation was observed between the percentage frequency of children using coping strategies and their perceived helpfulness. The Spearman correlation coefficient (r_s_) for the destructive coping strategy was − 0.429 (*p* < 0.001), whereas the r_s_ for the external coping strategy was − 0.170 (*p* = 0.032), and the r_s_ for the internal coping strategy was 0.165 (*p* = 0.033).

The test-retest reliability of the CFSS-DS scale indicated excellent agreement (ICC = 0.97). The children were most afraid of “choking” and having a stranger touch them (Table 4). The Kruskal‒Wallis test revealed no significant difference in PSS scores between the participants with different levels of dental fear: no clinical dental fear (nonclinical range; CFSS-DS score < 32; *n* = 25), borderline dental fear (borderline range; CFSS-DS score 32–38; *n* = 132), and clinical dental fear (clinical range; CFSS-DS score ≥ 39; *n* = 11) (Table 5).

**Table 4:** Frequency distribution of child fear survey Schedule-Dental subscale (CFSS-DS) scores

Item	Response				
	1	2	3	4	5
	N (%)	N (%)	N (%)	N (%)	N (%)
Dentists	61 (36.3)	87 (51.8)	20 (11.9)	-	-
Doctors	60 (35.7)	83 (49.4)	25 (14.9)	-	-
Injections	56 (33.3)	60 (35.7)	50 (29.8)	-	2 (1.2)
Having somebody examine your mouth	64 (38.1)	37 (22.0)	67 (39.9)	-	-
Having to open your mouth	75 (44.6)	65 (38.7)	28 (16.7)	-	-
Having stranger touch, you	3 (1.8)	18 (10.7)	59 (35.1)	39 (23.2)	49 (29.2)
Having somebody look at you	4 (2.4)	43 (25.6)	108 (64.3)	12 (7.1)	1 (0.6)
The dentist drilling	45 (26.6)	68 (40.5)	42 (25.0)	11 (6.5)	2 (1.2)
The sight of dentist drilling	43 (25.6)	53 (31.5)	69 (41.1)	1 (0.6)	2 (1.2)
The noise of dentist drilling	40 (23.8)	59 (35.1)	55 (32.7)	-	-
Having somebody put instruments in your mouth	45 (26.8)	68 (40.5)	55 (32.7)	-	-
Choking	-	-	4 (2.4)	28 (16.7)	136 (81.0)
Having to go to hospital	21 (12.5)	61 (36.3)	79 (47.0)	7 (4.2)	-
People in white uniforms	69 (41.1)	94 (56.0)	5 (3.0)	-	-
Having dentist clean your teeth	93 (55.4)	75 (44.6)	-	-	-

1 = Not afraid at all, 2 = A little bit, 3 = A fair amount of afraid, 4 = Pretty much afraid, 5 = Very afraid

**Table 5:** Comparison of parental stress scale scores based on the level of dental fear and behaviour of the child during dental treatment

Variable		*N* (%)	Mean PSS Score ± SD	Median (IQR)	H value	*p* value
Dental Fear	**CFSS** **-DS** ** ≥ ** **39**	11 (6.5)	34.73 ± 1.49	34.00 (2.00)	1.89	0.389
	**CFSS-DS 32–38**	132 (78.6)	35.54 ± 3.66	35.00 (2.00)		
	**CFSS-DS** ** < ** **32**	25 (14.9)	35.00 ± 4.67	36.00 (5.50)		
Behaviour	**Frankl 1**	8 (4.8)	35.23 ± 2.14	36.00 (3.00)	3.52	0.318
	**Frankl 2**	62 (36.9)	35.62 ± 4.65	35.00 (2.00)		
	**Frankl 3**	79 (47.0)	35.16 ± 2.78	34.00 (2.00)		
	**Frankl 4**	19 (11.3)	36.65 ± 2.67	36.00 (2.00)		

*SD* Standard deviation, *IQR* Interquartile range, *p* > 0.05 = not significant, indicating no significant difference in PSS scores for various levels of dental fear and the behaviour type, using Kruskal Wallis test

The inter-examiner reliability of the Frankl behaviour rating indicated almost perfect agreement (κ = 0.81), whereas the intra-examiner reliability for examiners 1 and 2 also showed almost perfect agreement for both examiners (κ = 0.98 and 0.95, respectively). The children’s behaviour did not vary significantly according to the type of treatment (χ² =14.40, *p* = 0.109) or the type of local anaesthesia administered (χ² =9.30, *p* = 0.157). There was no significant difference in the PSS scores based on the type of Frankl’s behaviour during dental treatment: rating 1-definitely negative: *n* = 8, rating 2-negative: *n* = 62, rating 3-positive: *n* = 79, and rating 4-definitely positive: *n* = 19 (Table 5). Frankl’s behaviour rating scale results revealed that 70 (48.7%) children had Frankl’s 1 and 2 peak behaviour ratings, categorised as uncooperative behaviour, and the remaining 98 (58.3%) had Frankl 3 and 4 peak behaviour ratings, categorised as cooperative behaviour. There was no significant difference in the PSS scores of parents of children with cooperative and uncooperative behaviour (Mann‒Whitney U = 3102.00 and *p* = 0.286).

The percentage of children using the three coping strategies, dental fear categories and behaviour types (dichotomised cooperative and uncooperative), along with demographic variables, were entered as independent variables into a generalised linear model, with PSS scores as the dependent variable. The results of the omnibus tests of the model coefficient were not significant (likelihood ratio = 6.27, *p* = 0.972), and none of the factors were significantly associated with the PSS scores of the parents. The adjusted odds ratio (aOR) was < 1 for age and sex of the parent and child, suggesting that parental stress decreases with increasing age of the child and parent, and if the parent or child is male, but these associations did not reach statistical significance (*p* > 0.05), indicating no meaningful association between the variables. Similarly, although the aOR was > 1 for dental fear and behaviour, indicating increased parental stress with increasing levels of fear (except for CFSS-DS ≥ 39) and uncooperative behaviour of the child, the associations of the PSS with dental fear and behaviour were not statistically significant (*p* > 0.05). The aORs for all the coping strategies were close to 1, indicating no association between the PSS stress scores and the coping strategies used by the child (Table 6).

**Table 6:** Generalised linear model with the mean PSS score as the dependent variable and the percentage frequency of coping strategies, dental fear levels and behaviour type of the child during dental treatment as the independent variables

Variable		aOR	95% Confidence Interval		*p* Value
			Lower	Upper	
Age (Child)		0.86	0.60	0.409	0.409
Sex (Child)	**Male (ref)**	0.96	0.30	0.939	0.939
	**Female**				
Age (Parent)		0.98	0.85	0.734	0.734
Sex (Parent)	**Male (ref)**	0.33	0.10	0.059	0.059
	**Female**				
Destructive Coping Strategies		0.99	0.97	0.915	0.915
External Coping Strategies		1.01	0.98	0.565	0.565
Internal Coping Strategies		1.01	0.98	0.584	0.584
CFSS-DS Score	**CFSS-DS < 32** **(ref)**				
	**CFSS-DS 32–38**	1.15	0.23	0.864	0.864
	**CFSS-DS ≥ 39**	0.59	0.03	0.721	0.721
Frankl’s Behaviour Rating	**Cooperative (ref)**	1.29	0.38	0.683	0.683
	**Uncooperative**				

*ref* reference category, *Cooperative* Frankl’s 3 and 4, *Uncooperative* Frankl’s 1 and 2, *aOR* Adjusted Odds Ratio, *p* > 0.05 = not significant, indicating no association of PSS scores with all the entered variables

## Discussion

We conducted the present study to investigate the role of parenting stress on the dental coping strategies, fear and behaviour of school-aged children. The concepts and questionnaire tools used in this study were based on the key concepts by Young et al. [7], Berry and Jones [21], Spirito et al. [25], and Cuthbert and Melamed [27], on stress, coping and dental fear assessment. All the included 8–12-year-old children had previous dental experience. Our study results suggest that parenting stress is not associated with the use of dental coping strategies, the degree of dental fear, or the type of dental behaviour of school-aged children who had undergone dental treatment earlier. The findings of the present study diverge from those of previous studies concerning the influence of parenting stress on children’s general behavioural outcomes. Parent‒child interactions significantly influence a child’s psychosocial development, including temperament, emotional adjustment, and behaviour regulation [17]. These interactions are shaped by parenting styles and stress levels. Parenting stress and child behaviour show reciprocal interactions [34]. Parenting stress is known to predict behaviour and emotional problems in school-aged children [19]. While high stress in parents of children with developmental disorders often stems from the child’s behavioural issues, stress in parents of typically developing children is influenced by factors such as depression, marital conflict, poor health, financial strain, and ineffective parenting [17, 35]. Parenting stress is linked to authoritarian style of parenting characterised by parental control, aggressive one-way communication and low levels of nurturance, whereas authoritative parenting, marked by warmth, consistent discipline, clarity in communication and nurturance, is associated with better child outcomes, such as reduced anxiety and greater social interaction [36]. Parenting practices can affect children’s social and cognitive development, which in turn affects the development of coping skills to manage the stress associated with dental treatment [37].

However, the parents of children between 8 and 12 years of age included in the present study experienced only mild stress [38], which explains the lack of a significant association between parenting stress and the study parameters. Parenting style has been identified as a significant determinant of dental anxiety and behavioural responses in preschool-aged children, whereas its influence appears to diminish during school-age years, particularly if they have previous dental experience [5]. Parental child-rearing attitudes, particularly self-sacrificial beliefs such as prioritising the child’s happiness at the parent’s expense, have been linked to children’s dental fear [39]. However, the mediating role of parenting on dental fear has been observed only during the initial visit, particularly in preschool-aged children. As the child gains his/her own dental experience and is exposed to the dentist’s behaviour guidance, the parent’s influence is diminished. It is possible that in older children, dental behaviour is more affected by the dentist’s behaviour management techniques or the child’s past negative dental experiences. Thus, the influence of parental factors on their child’s ability to cope during dental treatment procedures and consequently on their behaviour may diminish as the child grows older [39, 40]. Our study, which supports the abovementioned findings, revealed no significant associations of parenting stress with children’s dental fear, coping strategies, or behaviour in the dental setting. The children in our study were 8–12 years of age and had undergone previous dental treatment under local anaesthesia. Older children have greater maturity, a greater sense of independence and greater resilience, which improve their ability to cope with stressful situations such as dental treatment [40, 41]. The finding of a lack of association of parenting stress with dental fear and behaviour is similar to the finding of a lack of association of parental dental anxiety and parenting style with dental fear in school-aged children [5, 40]. The findings of our study underscore the importance of behaviour guidance techniques in dental situations, as the results are contrary to those of life situations, where parenting stress is a mediating factor between the effects of stressful life situations such as school problems or the illness of a family member and the child’s anxiety [42].

Children use coping strategies to address challenges such as pain and discomfort due to the disease, visit a hospital and establish communication with health care personnel [25]. Similarly, children employ coping strategies in dental settings to overcome dental fear and anxiety [9]. Typically, coping strategies can be related to comfort (having a parent near the child), distraction (listening to music with headphones), asking or telling the dentist or self-relaxation [43]. The coping strategies in the DCQ reflect similar coping strategies. The external and destructive coping strategies involve communication with the dentist or parents and are behavioural types, whereas the internal coping strategies are more cognitive types [9]. The coping strategies used by children during dental treatment are a process measure and do not reflect personality traits. Hence, variations in responses upon retesting are expected [25]. Thus, in this study, the reliability test kappa coefficients of the dental coping questionnaire showed a wider range.

A significant correlation is expected between the effectiveness of coping strategies and their use. Children are likely to use coping strategies that they have found effective [25]. Our study revealed a significant negative correlation, indicating that most participants struggled to employ effective coping strategies during dental treatment. This contrasts with earlier findings [9, 10], where children predominantly used internal coping mechanisms and found them beneficial. These differences can be attributed to variations in coping strategies across countries due to cultural differences [10]. In our study sample, children used more internal coping strategies, but they were perceived to be less helpful than destructive or external coping strategies were, which is in accordance with another study [29]. Additionally, the intellectual, social and emotional development of children and the influence of parents and siblings also affect the use of coping strategies [44]. However, the use of destructive coping strategies translates into uncooperative behaviour; hence, paediatric dentists need to guide child patients in practising appropriate coping strategies [26].

Children in school need help with coping strategies such as seeking emotional and social support from parents, peers or siblings, whereas adolescents prefer self-oriented coping strategies such as passive relaxation. As coping strategies vary with age, dental health professionals should guide child patients in selecting age-appropriate coping strategies. Talking about dental fear and telling the dentist in case of pain can be effective coping strategies [44]. Thus, encouraging children to vocalise triggers causing fear during dental treatment and suggesting strategies for perceived control would enhance their coping ability.

We entered demographic factors such as parents’ age and sex and children’s age and sex into the regression model, as the abovementioned factors can potentially influence dental fear and coping strategies. The presence of a parent can affect a child’s coping strategies during dental procedures [44], and parental dental anxiety can influence the child’s anxiety [41, 45]. As negative past experiences influence dental anxiety, dental anxiety can vary with age, leading to variation in parents’ dental anxiety with age [41]. Women are known to have more dental anxiety than men; thus, the accompanying father or mother may affect the child’s perception of dental fear differently [46, 47]. Children’s dental fear and ability to cope also vary with age and sex, with older children showing different levels of fear and coping strategies than younger children do [6, 10, 44]. Parenting stress also varies with age and gender. Mothers often perform multiple tasks involving childcare and housework and often face work‒family conflict [48]. However, given the changing role of fathers due to their greater involvement in childcare and education, no sex differences in parenting stress have been noted [49]. Younger parents can experience more parenting stress than middle-aged parents due to family, work and financial pressures [48, 49]. However, our results did not indicate variations in parenting stress based the age and sex of the accompanying parents.

School age is a critical period of lived experience in which a child learns to deal with mental and social challenges [19]. The development of dental fear and dental behavioural problems during this period can affect the ability of a child to deal with dental situations during adolescence and adulthood in the future [50]. An understanding of the factors associated with dental fear and behaviour will help dentists employ behaviour guidance strategies that increase children’s coping ability [31]. Hence, in this study, we focused on parenting stress, an underexplored factor, as a potential influence on children’s coping skills, dental fear, and dental behaviour during their school years. Although school-aged children are typically between 6 and 12 years of age, our study sample included children above 8 years of age, considering the cognitive abilities required to answer the DCQ and CFSS-DS questionnaires.

The findings of this cross-sectional study did not provide sufficient evidence to reject the null hypothesis, because of the lack of significant associations of parenting stress with dental coping strategies, dental fear, and the child’s behaviour in the dental clinic during treatment. The results suggest that parenting stress may not be a factor to be considered when assessing the factors affecting the coping strategies, fear and behaviour in dental clinics of 8–12-year-old children, with previous dental experience. When tailoring behaviour guidance strategies for older children, parental stress may not be a critical factor. Consequently, less emphasis may be placed on parental stress management and instead, the focus should be on well-established factors affecting dental fear and behaviour, such as the child’s individual characteristics and previous dental experiences. The above findings should be interpreted with caution due to the inherent limitations of the cross-sectional design, which precludes the establishment of causal relationships. In addition, participant responses may have been influenced by social desirability or recall bias, which could have affected the accuracy of self-reported measures. As the sample consisted of child–parent dyads from a single dental clinic, the findings may have limited generalisability. However, these findings may serve as valuable foundations for future large-scale investigations. Further investigations into factors such as the parents’ socioeconomic status, children’s previous dental experiences, temperament, parental dental anxiety, and parenting style are warranted to better understand how these factors, along with parenting stress, concomitantly affects children’s coping ability, dental fear and behaviour. Given the complex interplay of parenting and child-related variables, a person-centred analytical approach may offer deeper insights into these relationships.

## Conclusion

Within the limitations of the present study, it may be concluded that parenting stress does not significantly affect dental coping strategies or dental fear and behaviour during the dental treatment of children aged 8–12 years and with previous dental experience. Parenting stress may not be a critical factor affecting the school-aged children’s choice of coping strategies, fear or behaviour in dental situations and thus may not be essential to consider in behaviour guidance planning for their dental treatment.

## Data Availability

The data that support the findings of this study are available from the corresponding author upon reasonable request.

## Abbreviations

PSS: Parental stress scale
DCQ: Dental coping questionnaire
CFSS-DS: Children's Fear Survey Schedule-Dental Subscale
PS: Parenting stress subscale
LPS: Lack of parental satisfaction subscale
ICC: Intraclass correlation coefficient
SD: Standard deviation
IQR: Interquartile range
